# Successive Solvent Extraction, Characterization and Antioxidant Activities of Cardoon Waste (Leaves and Stems) Extracts: Comparative Study

**DOI:** 10.3390/molecules28031129

**Published:** 2023-01-23

**Authors:** Meryem Hajji Nabih, Hamza Boulika, Maryam El Hajam, Mohammed I. Alghonaim, Noureddine Idrissi Kandri, Sulaiman A. Alsalamah, Fehmi Boufahja

**Affiliations:** 1 Signals, Systems and Components Laboratory (SSC), Faculty of Sciences and Techniques, Sidi Mohamed Ben Abdellah University, Route Imouzzer, Atlas, Fez BP2202, Morocco; 2 Advanced Structures and Composites Center, University of Maine, Orono, ME 04469, USA; 3 Biology Department, College of Science, Imam Mohammad Ibn Saud Islamic University (IMSIU), Riyadh 11623, Saudi Arabia

**Keywords:** cardoon, Soxhlet, ultrasonic-assisted extraction, extraction, wastes, antioxidant, environment, Asteraceae

## Abstract

The main interest in the valorization of vegetable wastes is due to the peculiarity of their chemical composition in substances that present important properties. Among these substances, antioxidants could replace those industrially manufactured. In the present study, three solvents of different polarities (hexane, ethanol, and water) were applied for the extraction of phenolic compounds from *Cynara cardunculus* L. waste using two extraction methods: Soxhlet Extraction (SE) and Ultrasonic-Assisted Extraction (UAE). The obtained extracts were then characterized by Fourier-Transform Infrared (FTIR) spectroscopy and spectrophotometric determination of Total Phenolics (TPC), Total Flavonoids (TFC), and Condensed Tannins (CT). Total Antioxidant Capacity (TAC) and 2,2-diphenyl-1-picrylhydrazyl (DPPH) free radical scavenging activity of ethanol and water extracts of leaves and stems were also evaluated. High extraction yields were obtained by UAE. Water extracts had high yield regardless of the technique used for leaves and stems, and these extracts showed high TAC of 534.72 ± 3.83 mg AAE/g FM for leaves and 215.70 ± 8.87 mg AAE/g FM (mg of ascorbic acid equivalent per g of FM) for stems, and IC_50_ of 2077.491 μg/mL for leaves and 1248.185 μg/mL for stems. We explain the latter by the high total phenolic contents (TPCs), which reach 579.375 ± 3.662 mg GAE/g FM (mg of gallic acid equivalents per g of fresh matter) for leaves and 264.906 ± 3.500 mg GAE/g FM for stems. These results confirmed that the leaves and stems of the studied cardoon waste were, indeed, interesting sources of natural antioxidants.

## 1. Introduction

A vegetable plant in the Asteraceae family, cardoon (*Cynara Cardunculus L.*), has robust growth and is well adapted to Mediterranean climates due of its remarkable tolerance to a variety of climatic extremes, including high salt, soils with varying pH, and harsh temperatures [1]. It is also widespread in other countries, such as the United States, Australia, New Zealand, and Mexico [2]. The leaves are the largest component of the plant, making up around 35.4% of its dry weight, and they are a rich source of natural substances with anti-inflammatory and antibacterial properties [3]. This makes them very useful, for example, in the food and pharmaceutical industries in the preparation of certain cheeses, paper pulp, edible oils, bioenergy, as well as fodder [3]. The numerous industrial uses of cardoon are essential to its exploitation and economic value. However, the consumption of food and industrial processes result in millions of tons of garbage [4]. They are frequently used as fuel, generating polluting products and harming the environmental balance. It is, therefore, essential to find new alternatives for the recovery of this waste. Their diversified chemical compositions can become a potential resource of high-value-added raw materials and can be potential precursors for the preparation of environmental adsorbents for reductions in or even removal of organic dyes [5], in addition to being a significant source of biologically active molecules, such as antioxidants [6], the latter of which is regulated by a number of variables, including the section of the plant, the cultivation soil, and the maturity stage [1]. The increasing demand for these components prompted researchers to quantify phenolic compounds using an affordable and ecologically friendly extraction approach and to assess their antioxidant properties. Studies have shown that phenolic chemicals found in food wastes, including grape pomace, coffee ground waste, and orange peels, have antioxidant properties [7] and these leftovers might be utilised to develop new, healthy dietary supplements. In the numerous industrial applications of phenolic compounds, the efficiency of the extraction process and the polarity of the solvents used are observed [8]. Therefore, extraction techniques remain an essential process for the recovery of phenolic compounds from plant by-products. Several conventional extraction methods, such as maceration and Soxhlet, have used simple techniques for the extraction of specific compounds. These extracts can either be used directly or added to formulations as herbal medicines. These techniques generally take a long time and require higher solvent volumes. In recent years, more experiments have been conducted at the laboratory scale using these green extraction techniques that are not applicable in the industrial sector. These green extraction techniques have been used to obtain valuable extracts from plant samples. Non-conventional extraction techniques provide higher and more selective recovery of products. These unconventional techniques include microwaves, ultrasound, supercritical fluids, high-pressure liquids, pulsed electric fields, and enzyme-assisted extraction techniques [9]. In the present study, two extraction methods are used, one conventional and the other non-conventional, for the extraction of phenolic compounds from *C. cardunculus* waste, Soxhlet Extraction (SE) and Ultrasonic-Assisted Extraction (UAE), using three solvents of various polarities (Hexane, Ethanol, Water). Qualitative and quantitative characterization of the extracts obtained was performed by Fourier-Transform Infrared (FTIR) spectroscopy and spectrophotometric determination of total phenolics (TPC), total flavonoids (TFC), and condensed tannins (CT). Total Antioxidant Capacity (TAC) and 2,2-diphenyl-1-picrylhydrazyl (DPPH) free radical scavenging activity of ethanol and water extracts of leaves and stems were also evaluated.

## 2. Results and Discussion

### 2.1. Characterization

#### 2.1.1. Yield

The obtained yields with distilled water and ethanol are important whatever the used extraction technique for both leaves and stems (Table 1). It is also observed that the yield of a leaf extract is higher than that of stem extract for both methods, with values ranging from 16.37 ± 0.46 to 29.40 ± 1.47% for Soxhlet Extraction (SE) and values between 24.00 ± 2.36% and 33.00 ± 4.98% for Ultrasonic-Assisted Extraction (UAE). Extraction by UAE gave higher yields for both leaf and stem extracts compared to the SE method. It is also noted that hexane gave a lower yield compared to other solvents, which is in a range of 1.93 ± 0.85% and 13.00 ± 1.87%. Similar results were reported for the extracts from cardoon leaves by ultrasound with an average of 50% [3].

#### 2.1.2. FTIR Spectroscopy

**Figure 1:**
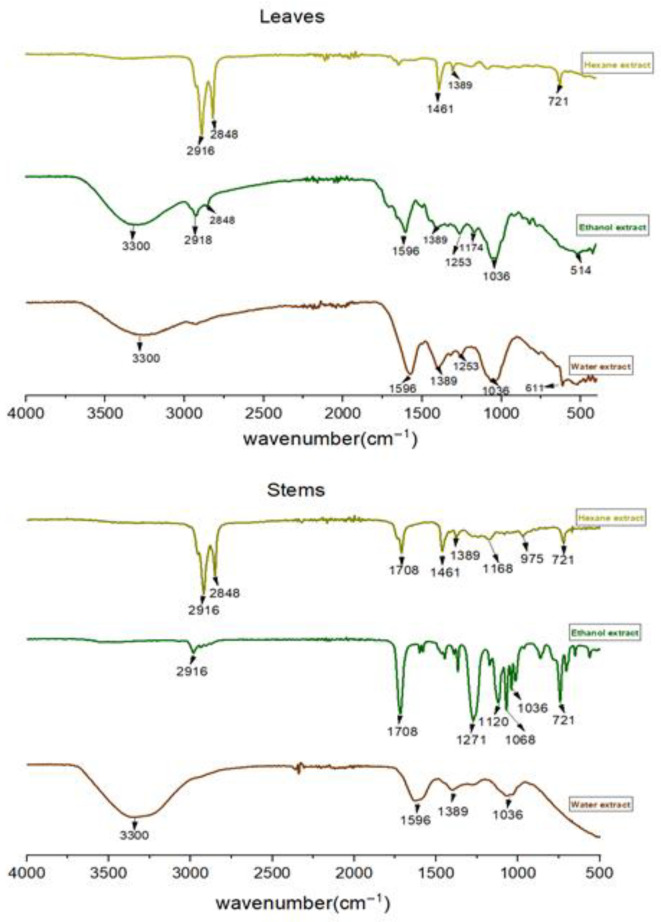
Fourier-Transform Infrared (FTIR) spectra of hexane, ethanol, and water extracts for leaves and stems.

FTIR analysis allows for the identification of the functional groups of the obtained extracts. The absorption spectra of the hexane, ethanol, and water extracts for leaves and stems are gathered in Figure 1 and their wavenumbers and assignments are grouped in Table 2. Infrared absorption spectra of leaves and stems for ethanol and water extracts have similar appearances with almost the same absorption bands, with a slight difference in intensities. Indeed, the hexane extracts show a difference in the majority of adsorption bands with those extracted by ethanol and water. For the spectra of ethanol and water extracts, a broad and intense band at 3300 cm^−1^ is shown, which corresponds to the elongation vibration of the O-H hydroxyl group [10] of the alcohol; this band reveals the presence of flavonoids and tannins [11]. The absorption bands around 2918 and 2916 cm^−1^ are characteristic of the elongation vibration of the O-H groups of carboxylic acids and, thus, the extracts containing anthocyanosides, hydrolyzable tannins, and carotenoids [11]. The absorption band around 1596 cm^−1^ indicates the presence of aromatic C=C bonds. The bands observed at 1389 cm^−1^ and 1036 cm^−1^ would represent the C-O functional groups of ether oxide and C-N of amines, respectively; analysis of these data revealed that these extracts contain flavanols [11]. The low-intensity band at 1253 cm^−1^ is due to the elongation vibrations associated with C-O bonds. The peaks between 1000 and 500 cm^−1^ highlight the presence of C-H bonds with a medium intensity. For the hexane extracts for the leaves, it is observed that they have the same functional grouping as their stems. We observe an absorption band around 2916 cm^−1^, which is associated with the elongation vibration of the O-H groups of carboxylic acids. The band located at 2848 cm^−1^ corresponds to the elongation vibration of the aromatic C-H bands of alkaloids [12]. We also observe the presence of peaks at 1461 cm^−1^ and 1389 cm^−1^, indicating the presence of C=O stretching and N-H bending of the carboxylic acid bond (presence of alkaloids, tannins, flavonoids, glycosides, and saponins) [13]. The band located at 1708 cm^−1^ is characteristic of elongation of the bond C=C of the alkene function [14]. The peak at 721 cm^−1^ shows the presence of a C-N group [15,16]. 

For all three extracts, a range between 700 and 1500 cm^−1^ is observed; the spectra observed in this area show many clear bands, which are mainly attributed to deformation and stretching vibrations of the alkaloid ring system [12].

The spectra clearly show the phenolic nature of all the extracts obtained in this work [17].

#### 2.1.3. Spectrophotometric Determination

**a.** TPCs

Table 3 collects the TPCs of leaf and stem extracts of cardoon waste. They range from 32.472 ± 3.988 to 579.375 ± 3.662 mg GAE/g FM (mg of gallic acid equivalents per g of fresh matter) for leaves and from 25.392 ± 4.313 to 264.906 ± 3.500 mg GAE/g FM for stems. The TPCs of the water extracts of leaves and stems are higher than those obtained by ethanol and hexane with a content of 579.375 ± 3.662 mg GAE/g FM and 264.906 ± 3.500 mg GAE/g FM, respectively. These results differ for ethanol extracts obtained from cardoon leaves by maceration: phenolic contents ranged from 86.8 ± 3.5 mg GAE/L (corresponding to 140 mg GAE/100 g fresh leaves weight) to 147.2175 ± 4.4 mg GAE/L (corresponding to 230 mg GAE/100 g fresh leaves weight) [2].

**b.** TFCs

The water extracts of leaves and stems contain TFCs in the order of 5.1204 ± 0.361 and 5.0237 ± 0.386 mg QE/g FM (mg of quercetin equivalent per g of FM), respectively. Lower contents are present in ethanol extracts of 2.8624 ± 0.264 mg QE/g FM for leaves and 2.9323 ± 0.357 mg QE/g FM for stems. TFCs were present in trace amounts in hexane extracts (Table 3). These results are similar to those obtained by previous studies, which showed that TFCs ranged from 7.93 ± 0.43 to 4.10 ± 0.73 mg CE/g DM (mg of catechin equivalents per g of dry matter) [18].

**c.** CTs

The CTs found in the water extracts of leaves and stems are in the order of 18.446 ± 4.674 and 18.166 ± 4.747 mg CE/g FM (mg of catechin equivalents per g of FM), respectively (Table 3). A lower content is found in ethanol extracts in the order of 11.912 ± 4.394 mg CE/g FM for leaves and in the order of 12.115 ± 4.664 mg CE/g FM for stems. The CTs in the hexane extracts for leaves and stems are lower with contents of 0.302 ± 0.010 mg EC/g FM and 0.982 ± 0.025 mg EC/g FM, respectively. These results are different to those obtained from *Vitis vinifera* L leaf extracts, which showed that CTs vary from 5.30 ± 0.04 to 5.14 ± 0.16 mg CE/g DM (mg of catechin equivalents per g of DM), depending on the region [18].

#### 2.1.4. TAC

From the obtained results (Table 4), it is observed that the water extracts show a higher activity for leaves and stems of 534.72 ± 3.83 and 215.70 ± 8.87 mg AAE/g FM (mg of ascorbic acid equivalent per g of FM), respectively; on the other hand, the ethanol extracts show a lower activity of 84.76 ± 3.83 mg AAE/g FM for leaves and 179.41 ± 11.09 mg AAE/g FM for stems. These results indicated that the extracts of the leaves and stems of cardoon had the ability to transform reactive radical species into more stable ones, as in [19]. These results are totally different to those found by Selka et al., who showed that the total antioxidant capacity of *Vitis vinifera L*. leaves is in a range of 20.545 ± 3.961, 18.128 ± 0.157 and 10.37 ± 0.03 mg ascorbic acid Eq/g dry matter, depending on the region [18]. 

#### 2.1.5. DPPH Free Radical Scavenging Activity

**Figure 2:**
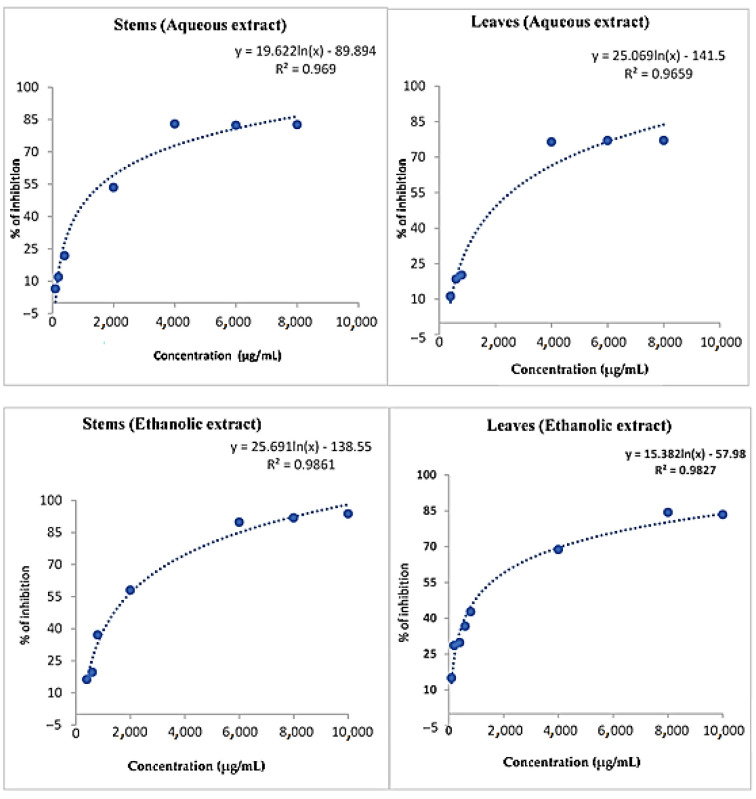
DPPH free radical scavenging activity curves of water and ethanol extracts of leaves and stems.

The extracts from leaves and stems significantly inhibited DPPH radicals at different concentrations (Figure 2). The free radical scavenging capacity increased with increasing free radical concentration. The stem extracts showed higher DPPH free radical scavenging activity than the leaf extracts, with a percentage inhibition of 82.63 ± 0.700% at 8000 µg/mL for the water extracts, while the ethanol extract achieved a percentage inhibition of 91.83 ± 0.062% at the same concentration (Table 5). Water and ethanol extracts of leaves reached 77.10 ± 0.210% and 84.33 ± 0.061%, respectively, at 8000 µg/mL concentration. Butylated hydroxytoluene (BHT) used as a standard inhibited 76.78 ± 0.001% of DPPH free radicals at 500 µg/mL. These results are different from those found by S. I. M. Dieng et al. who showed that the extract of *Piliostigma thonningii* (Schumach.) Milne-Redh. reaches almost its maximum activity at 125 µg/mL, with a percentage of inhibition of 91.11 ± 0.59%. As for the leaf extract, its highest inhibition percentage, 75.04 ± 0.91%, is observed at a concentration of 250 µg/mL [20].

**Figure 3:**
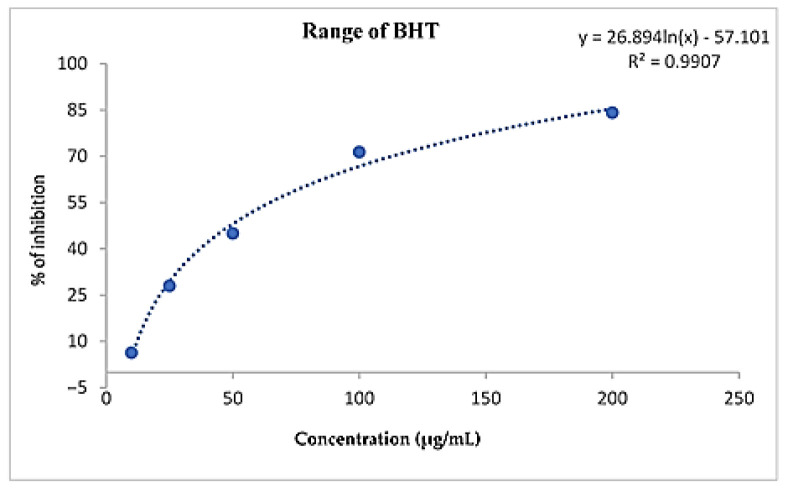
DPPH free radical scavenging activity curves for butylated hydroxytoluene (BHT).

For a better comparison of the activities of the different extracts tested, the concentration of the extract ensuring 50% radical scavenging (IC_50_) was determined graphically by linear regression (Table 6). The extracts showed optimum antioxidant activity from 1118.667 to 2077.491 µg/mL for leaves and from 1248.185 to 1539.396 µg/mL for stems. The ethanol leaf extract showed better radical scavenging activity, with the smallest IC_50_ value (1118.667 µg/mL); however, the distilled water leaf extract showed relatively lower IC_50_ free radical scavenging activity of 2077.491 µg/mL. The ethanol stem extract also showed better radical scavenging activity, with an IC_50_ value of 1248.185 µg/mL; on the other hand, the water extracts give an IC_50_ value of 1539.396 µg/mL. BHT showed an IC_50_ equal to 53.642 µg/mL (Figure 3). These results were different to those obtained by extracts from the leaves of *Piliostigma thonningii* (Schumach.) Milne-Redh. leaf extracts, indicating that the concentration of the extract providing 50% radical scavenging is 109 ± 6.25 µg/mL [20].

## 3. Materials and Methods

### 3.1. Chemical Products

The different chemicals used in this study are:

Distilled water, ethanol, hexane, methanol, Folin-Ciocalteu reagent, hydrochloric acid, quercetin acid, gallic acid, glacial acetic acid, sodium carbonate, aluminum chloride, catechin, vanillin acid reagent, 2,2-diphenyl-1-picrylhydrazyl, butylated hydroxytoluene, molybdate, and ascorbic acid.

### 3.2. Equipment

The different equipment used in this study and its brands are listed in Table 7:

### 3.3. Plant Material

In Morocco, cardoon waste (leaves and stems) was collected from a local food market in the city of Fez. The waste was washed with water, dried in the sun, and then separated into leaves and stems. The dried leaves and stems were ground separately using an IKA automatic tube grinder and then sieved. The obtained powders were characterized by inductively coupled plasma atomic emission spectroscopy (ICP-AES), scanning electron microscopy coupled to EDX (SEM/EDX), X-ray diffraction (XRD), Fourier-transform infrared spectroscopy (FTIR), thermogravimetric analysis (TGA/DTA), and preliminary analyses according to [21]; these powders were subjected to a successive extraction procedure.

#### Successive Extraction

The obtained powders separately undergo successive extraction with hexane, ethanol, and then water in the order of increasing polarity, using two methods of extraction: Soxhlet Extraction and Ultrasonic-Assisted Extraction. 

SE: Thus, 6 g of powder was put in a cartridge, the whole was placed inside the Soxhlet-type apparatus, and 200 mL of the used solvent was poured into a flask. The extraction time was 6 h, and the obtained extracts were placed in a refrigerator at 4 °C [22].

UAE: Here, 6 g of powder was dispersed in 200 mL of the used solvent and then subjected to UAE at room temperature, a frequency of 35 kHz, and a power of 100 W. The extraction was performed in two cycles of 15 min. After each cycle, the samples were shaken with a vortex for 1 min. The obtained extract was rested for 15 min at room temperature, then the plant powder was separated from the liquid by vacuum filtration and the obtained filtrate was stored at 4 °C [3].

### 3.4. Physicochemical and Phytochemical Characterizations

#### 3.4.1. FTIR Spectroscopy 

A Bruker Vertex 70 FTIR Spectroscopy in Attenuated Total Reflectance (ATR) mode was used to identify the functional groups that were present in the extracts. The resolution of the FTIR spectra, which range in wavelength from 4000 to 500 cm^−1^, is 4 cm^−1^.

#### 3.4.2. Total Phenolic Content (TPC)

With a slight modification, the method used by El-Guendouz et al. [23] was used to determine the TPC. After placing 50 μL of each extract and the standard (gallic acid at various concentrations) in separate test tubes, 450 μL of the 10% Folin–Ciocalteu reagent was added, followed by 450 μL of sodium carbonate (7.5%) after 8 min. The absorbance at 760 nm was measured following a 2 h incubation period at room temperature. 

#### 3.4.3. Total Flavonoid Content (TFC) 

The method of El-Guendouz et al. [23] was used to determine the amount of flavonoids. A volume of 500 μL of aluminum chloride solution (2%) (2 g of aluminum chloride in 100 mL of 5% *v/v* glacial acetic acid solution in methanol) was placed in test tubes and 500 μL of each extract and standard (quercetin at different concentrations) was added separately. The absorbance was measured at 420 nm after 1 h of incubation at room temperature.

#### 3.4.4. Condensed Tannins (CT)

The slightly modified vanillic acid method was used to quantify the amount of CT [24]. Each test tube was filled with 50 µL of each extract and the standard (different amounts of the catechin solution) separately, and 3 mL of the vanillin/methanol solution (4% *w/v*) was added to each test tube. The mixtures were stirred manually, and each tube received 1.5 mL of concentrated hydrochloric acid. The resulting mixtures were allowed to react for 15 min at room temperature in the dark. The absorbance was measured at 500 nm.

#### 3.4.5. Total Antioxidant Capacity (TAC) 

Here, 1.5 mL of molybdate solution was mixed in test tubes with 50 µL of each extract separately. The tubes were incubated at 95 °C for 90 min. The absorbance was determined at 695 nm. A calibration range was created from a series of dilutions of an ascorbic acid (AA) stock solution [25].

#### 3.4.6. 2,2-.diphenyl-1-picrylhydrazyl (DPPH) Radical Scavenging Assay

The Brand-Williams method was used to determine the ability of the extracts to scavenge free radicals [26]. Thus, 1 mL of an ethanolic solution (60 M) of DPPH was mixed with 25 µL of each extract at different dilutions. The absorbance was measured at 515 nm after 60 min of incubation at room temperature. As a negative control, the absorbance of a blank sample containing the same volume of ethanol and DPPH solution was measured. Butylated hydroxytoluene (BHT) was used as a reference. The following equation was used to determine the percentage of free radical scavenging activity of each extract that was inhibited:

(1) I%=(Absc−Abst)Absc×100

With *I*%: percentage of inhibition of the antioxidant activity 

*Abs_c_*: absorbance of the blank 

*Abs_t_*: absorbance of the samples.

## 4. Conclusions

This study aimed at the valorization of cardoon waste through the extraction of its active principles via Soxhlet Extraction (SE) and Ultrasonic-Assisted Extraction (UAE). The obtained extraction yields using three different solvents revealed interesting yields of extract for the extraction by UAE, with an overall yield of 76.4 ± 8.49% for leaves and 55.6 ± 6.07% for stems. It is also observed that distilled water and ethanol give a significant yield regardless of the extraction technique used for leaves and stems. The yield of the leaf extract is higher than that of the stem extract for both techniques. The phenolic extracts were qualitatively characterized by FTIR spectroscopy; the results obtained clearly show that the spectra of the extracts studied in this work have a phenolic nature. Quantitative analysis of the extracts by spectrophotometric determination of total phenolics, total flavonoids, and condensed tannins, respectively, reveals the TPCs of the extracts of cardoon waste leaves and stems. They ranged from 32.472 ± 3.988 to 579.375 ± 3.662 mg GAE/g FM (mg of gallic acid equivalents per g of fresh matter) for leaves and from 25.392 ± 4.313 to 264.906 ± 3.500 mg GAE/g FM for stems. TFCs for leaf extracts ranged from 0.200 ± 0.005 to 5.1204 ± 0.361 mg QE/g FM (mg of quercetin equivalent per g of fresh matter) and for stems from 0.201 ± 0.016 to 5.0237 ± 0.386 mg QE/g FM. CTs ranged from 0.302 ± 0.010 to 18.446 ± 4.674 mg CE/g FM (mg of catechin equivalents per g of fresh matter) for leaves and from 0.982 ± 0.025 to 18.166 ± 4.747 mg CE/g FM for stems. The water extracts showed high TAC of 534.72 mg AAE/g FM (mg of ascorbic acid equivalent per g of fresh matter) for leaves and 215.70 mg AAE/g FM for stems. On the other hand, the ethanol extracts show lower activity, with a content of 84.76 mg AAE/g FM for leaves and 179.41 mg AAE/g FM for stems. These results indicate that the extracts from the leaves and stems of cardoon have an ability to transform reactive radical species into more stable species. The DPPH free radical scavenging activity of all extracts showed that leaves and stems significantly inhibited DPPH radicals at different concentrations; it is also observed that the free radical scavenging capacity increases with the concentration of extracts. The concentration of the extract ensuring 50% radical scavenging IC_50_ is 2077.491 μg/mL for leaves and 1248.185 μg/mL for stems. We explain the latter by the high TPCs. These results confirmed that the leaves and stems of the studied cardoon waste were, indeed, interesting sources of natural antioxidants. According to all these results, we can consider further valorization of these wastes in applications, such as the depollution of wastewater by adsorption of organic dyes or metals, the inhibition of metal oxidation, and the synthesis of nanocomposite materials.

## Figures and Tables

**Table 1:** Extraction yields of phenolic compounds from leaves and stems of *Cynara cardunculus* waste.

Solvent	Yield%			
	Soxhlet Extraction		Ultrasonic Assisted Extraction	
	Leaves	Stems	Leaves	Stems
Hexane	1.93 ± 0.85	1.95 ± 0.06	19.40 ± 1.15	13.00 ± 1.87
Ethanol	16.37 ± 0.46	15.20 ± 1.32	24.00 ± 2.36	17.20 ± 0.95
Distilled water	29.40 ± 1.47	24.51 ± 0.86	33.00 ± 4.98	25.40 ± 3.25
Total	47.7 ± 2.78	41.66 ± 2.24	76.4 ± 8.49	55.6 ± 6.07

**Table 2:** Assignment of the absorption bands of the Fourier-Transform Infrared (FTIR) spectra of the extracts.

Hexane Extract		Ethanol Extract		Water Extract	
Wavenumber (cm^−1^)	Assignment	Wavenumber (cm^−1^)	Assignment	Wavenumber (cm^−1^)	Assignment
2916	O-H (carboxylic acid)	3300	O-H (alcohol)	3300	O-H (alcohol)
2848	C-H (elongation)	2918–2916	O-H (carboxylic acid)	1596	C=C (aromatic cycle)
1708	C=C (alcene)	1708	C=C (alcene)	1389	C-O (ether oxide)
1461	C=O (elongation)	1596	C=C (aromatic cycle)	1253	C-O
1389	N-H (deformation)	1389	C-O (ether oxide)	1036	C-N (amines)
1168	C-N (elongation)	1253	C-O	1000–500	C-H
721	C-N (deformation)	1036	C-N (amines)	-	-
-	-	1000–500	C-H	-	-

**Table 3:** Total phenolic, total flavonoid, and condensed tannin of cardoon waste extracts from Ultrasonic-Assisted Extraction method.

Samples	Phenolics (mg GAE/g FM)		Flavonoids (mg QE/g FM)	Condensed Tannins (mg CE/g FM)
Leaves	Hexane extract	32.472 ± 3.988	0.200 ± 0.005	0.302 ± 0.010
	Ethanol extract	82.524 ± 10.661	2.8624 ± 0.264	11.912 ± 4.394
	Water extract	579.375 ± 3.662	5.1204 ± 0.361	18.446 ± 4.674
Stems	Hexane extract	25.392 ± 4.313	0.201 ± 0.016	0.982 ± 0.025
	Ethanol extract	108.404 ± 3.174	2.9323 ± 0.357	12.115 ± 4.664
	Water extract	264.906 ± 3.500	5.0237 ± 0.386	18.166 ± 4.747

**Table 4:** Total antioxidant capacity (TAC) of water and ethanol extracts of leaves and stems.

Samples		TAC (mg Ascorbic Acid Equivalents/g Fresh Matter)
Leaves	Ethanol extract	84.76 ± 3.83
	Water extract	534.72 ± 3.83
Stems	Ethanol extract	179.41 ± 11.09
	Water extract	215.70 ± 8.87

**Table 5:** Percentage of DPPH free radical inhibition of water and ethanol extracts of leaves and stems.

Concentration (µg/mL)	% of Inhibition			
	Water Extract		Ethanol Extract	
	Leaves	Stems	Leaves	Stems
8000	77.10 ± 0.210	82.63 ± 0.700	84.33 ± 0.061	91.83 ± 0.062
	butylated hydroxytoluene (BHT)			
500	76.78 ± 0.001			

**Table 6:** Half-maximal inhibitory concentration (IC_50_) of the 2,2-diphenyl-1-picrylhydrazyl (DPPH) by water and ethanol extracts and butylated hydroxytoluene (BHT).

Samples		DPPH Scavenging IC50 (μg/mL)
Range of BHT		53.642
Leaves	Ethanol extract	1118.667
	Water extract	2077.491
Steams	Ethanol extract	1539.396
	Water extract	1248.185

**Table 7:** Equipment used in this study.

Equipment	Brand Name
Automatic Mill	IKA tube Mill control
Oven	Binder
UV Spectrophotometer	Biobase
Spectrometer	FTIR Bruker Vertex 70
Soxhlet Extractor	Heating Mantle
Ultrasound	Elma
Electronic Balance	Nahita
Rotavapor	Büchi R-114
Refrigerator	Siera
Cartridge Glasses	-

## Data Availability

The data cannot be shared due to restrictions of privacy and regulation.

## Sample Availability

Samples of the compounds are not available from the authors.
